# Choice of Moisturiser for Eczema Treatment (COMET): study protocol for a randomized controlled trial

**DOI:** 10.1186/s13063-015-0830-y

**Published:** 2015-07-15

**Authors:** Matthew J Ridd, Niamh M Redmond, Sandra Hollinghurst, Nicola Ball, Lindsay Shaw, Richard Guy, Victoria Wilson, Chris Metcalfe, Sarah Purdy

**Affiliations:** School of Social & Community Medicine, University of Bristol, 39 Whatley Road, Bristol, BS8 2PS UK; Department of Dermatology, University Hospitals Bristol NHS Foundation Trust, Marlborough Street, Bristol, BS1 3NU UK; Department of Pharmacy & Pharmacology, University of Bath, Claverton Down, Bath, BA2 7AY UK; Bristol Randomised Trials Collaboration, University of Bristol, 39 Whatley Road, Bristol, BS8 2PS UK

**Keywords:** children, eczema, emollients, feasibility, primary care, RCT

## Abstract

**Background:**

Eczema is common in children and in the UK most cases are managed in primary care. The foundation of all treatment is the regular use of leave-on emollients to preserve and restore moisture to the skin. This not only improves comfort but may also reduce the need for rescue treatment for ‘flares’, such as topical corticosteroids. However, clinicians can prescribe many different types of emollient and there is a paucity of evidence to guide this choice. One reason for this may be the challenges of conducting a clinical trial: are parents or carers of young children willing to be randomly allocated an emollient and followed up for a meaningful amount of time?

**Design:**

This is a single-centre feasibility study of a pragmatic, four-arm, single-masked, randomized trial. Children with eczema who are eligible (from 1 month to less than 5 years of age, not known to be sensitive or allergic to any of study emollients or their constituents) are recruited via their general practices. Participants are allocated Aveeno® lotion, Diprobase® cream, Doublebase® gel or Hydromol® ointment via a web-based system, using a simple randomization process in a 1:1:1:1 fashion. Researchers are masked to the study emollient. Participants are assessed at baseline and followed up for 3 months. Data are collected by daily diaries, monthly researcher visits and review of electronic medical records. Because this is a feasibility study, a formal sample size calculation for the estimation of treatment effectiveness has not be made but we aim to recruit 160 participants.

**Discussion:**

Recruitment is on-going. At the end of the study, as well as being able to answer the question, ‘Is it is possible to recruit and retain children with eczema from primary care into a four-arm randomized trial of emollients?’, we will also have collected important data on the acceptability and effectiveness of four commonly used emollients.

**Trial registration:**

Current Controlled Trials ISRCTN21828118 and Clinical Trials Register EudraCT2013-003001-26.

**Electronic supplementary material:**

The online version of this article (doi:10.1186/s13063-015-0830-y) contains supplementary material, which is available to authorized users.

## Background

Childhood eczema is the most common inflammatory skin disease, affecting around 20% of children in the UK, with the peak incidence in the first two years of life [1]. Eczema, characterized by dry and itchy skin, can have a significant impact on the quality of the affected child’s life and that of the child’s family – the more severe the eczema, the greater the effect [2]. The resulting impairment in health-related quality of life is comparable to that of other chronic diseases of childhood, including diabetes and asthma [3]. Eczema also places a significant financial burden on both families and the National Health Service (NHS) [4]. In the UK, the majority of children with eczema are diagnosed and their treatment is managed exclusively by their general practitioners (GPs) [5].

The cornerstone of eczema treatment in all settings is the regular application of ‘leave-on’ emollients, which rehydrate and improve the comfort of dry skin [6]. There is also some evidence to suggest that emollients reduce the need for topical corticosteroids [7], which are used to treat disease ‘flares’ but which can have harmful effects if applied inappropriately. Clinicians in primary care can prescribe a wide variety of emollients, which come in different formulations (lotions, creams, gels and ointments), varying in consistency from ‘light’ to ‘heavy’. Light preparations (creams and lotions) have a high water content, are absorbed quickly and are more cosmetically acceptable but may require more frequent application. Heavy preparations (ointments) have a longerlasting effect, owing to their high oil content, but as a consequence make the skin feel greasy.

There is weak and limited research evidence to guide which emollient should be prescribed and how often it should be applied [8]. The last systematic review of eczema treatments was published in 2000 and concluded that there was ‘a virtual absence of clinically useful randomized controlled trials data on the use of emollients in atopic eczema’ [9], with five published randomized controlled trials being criticized for poor quality of reporting, poor analysis or short duration. Subsequently, the 2007 NICE guidelines on the management of atopic eczema [10] and the GREAT database (an on-line repository of trials in eczema) [11] have not identified any significant new studies of emollients in eczema.

It is therefore unsurprising that different clinicians prescribe different emollients, leading to emollients being only partly used (or not at all) and multiple consultations before parents or carers are recommended an emollient that works for their child. Indeed, some families may ‘give up’ and turn to less orthodox treatments, which may be harmful [12]. This is important to the NHS because, in addition to the cost of wasted medicines and repeat consultations, it may be that the older, cheaper emollients are as effective as, or better than, newer, more expensive ones. Sound data are also required to support the belief that regular use of emollients reduces exacerbations of eczema and topical corticosteroid use. It is an issue of importance to both patients and doctors: “Which emollients are the most effective and safe in treating eczema?” emerged as one of the highest priorities for further research in a recent research priority setting exercise [13].

Therefore, there is a need for robust randomized trial evidence with regards to the most clinically and cost-effective emollients for eczema. However, there are a number of challenges in undertaking such a study. First, choosing which emollients should be compared – the British National Formulary for Children [14] lists over 30 alternatives. Second, establishing the optimal means of recruiting children into the study and ensuring that short and medium-term outcome data on effectiveness and cost can be collected. Third, choosing the most appropriate primary outcome measure. To our knowledge, no study has attempted to address these key questions in primary care.

The aim of the Choice of Moisturiser for Eczema Treatment (COMET) study is to determine whether a clinical trial comparing four different emollients for the treatment of childhood eczema can be conducted and to obtain important information that will inform the design and size of a definitive trial.

## Methods

### Design

COMET is a feasibility study of a pragmatic, single-masked, randomized clinical trial to compare the clinical benefit and cost-effectiveness of leave-on emollients in the treatment of children with eczema in primary care. The aim is to recruit 160 children, randomly allocate them to one of four emollients and follow them up for 3 months. The primary means of data collection are parent-completed daily diaries, monthly assessments, and retrospective review of electronic medical records.

### Setting

General practices located in Bristol, South Gloucestershire and North Somerset will be invited to participate in the trial via the West of England Clinical Research Network.. We aim to recruit practices from a diversity of settings, using different computer systems and with varying levels of research activity and experience. Criteria for entry into the trial include having at least one GP trained in good clinical practice and a willingness to undertake patient mail-outs and prescribe the study emollients.

### Participants

Children are eligible if they: are aged from 1 month to under 5 years; have eczema (diagnosed by a doctor or an appropriately qualified health care professional with oversight from a medically qualified doctor); and are not known to be sensitive or allergic to any of the study emollients or their constituents. An adult with parental responsibility must consent for the child to participate. Throughout this paper, the term ‘parent’ will be used to denote all carers or guardians.

Eligibility will be confirmed by an appropriately qualified health care professional for patients referred by the practice, with oversight from a medically qualified doctor, or by the chief investigator (or his deputy) for patients who self-refer. All those making eligibility decisions will be trained in good clinical practice.

### Recruitment

There are two recruitment methods: ‘self-referral’ or ‘in consultation’.

‘Self-referral’ is defined as when parents of potentially eligible children are invited to contact the research team directly by means of a practice mailshot, practice waiting room posters or study flyers. Parents who respond have their child’s eligibility and understanding of the study checked by a researcher. Once confirmed, written informed consent is received at the baseline visit.

‘In consultation’ is defined as when parents of eligible children are recruited by a GP or practice nurse during a surgery visit (which need not be for the child’s eczema). The GP or practice nurse establishes eligibility, receives written informed consent and conducts the randomization. Practice nurses and GPs are asked to record all approaches to potentially eligible participants in a recruitment log.

### Intervention and assignment of intervention

Participants are randomly allocated to one of four emollients (Aveeno® lotion 400 ml, Diprobase® cream 500 g, Doublebase® gel 500 g, Hydromol® ointment 500g) to use as their primary leave-on emollient with directions to ‘Use twice daily and when required.’ These were chosen because they represent each of the different formulations (lotion, cream, gel and ointment), are among the most commonly prescribed and vary in cost. In addition, Doublebase® and Diprobase® emerged as the most popular in a patient preference study and mechanistic studies have shown that these emollients enhance skin barrier function (Professor Hywel Williams, personal communication). Allocation is done by the Bristol Randomised Trials Collaboration web-based system, using a simple randomization process in a 1:1:1:1 allocation.

All study emollients are prescribed for the duration of the study by the participant’s GP surgery as per normal care. For children recruited via the self-referral route, randomization is conducted by the trial coordinator (or the trial coordinator’s deputy) upon confirmation of eligibility and consent, who will then contact the practice to arrange prescription of the study emollient. For children recruited in consultation, the recruiting GP or practice nurse is responsible for randomization and prescribes the allocated emollient at that visit. This process is kept secret from the researchers who perform the assessment visits.

Therefore COMET is a single-masked study, where clinician, parent and trial coordinator are unmasked but the researchers undertaking the baseline and follow-up visits are masked. This is to ensure that the ‘objective’ assessments of eczema severity are unbiased, that is are not influenced by researcher knowledge of the type of emollient being used. To maintain masking, the following steps will be taken. First, researchers undertaking the assessments do not have access to the randomization system and are not able to identify which emollient has been assigned to which participant. Second, clinicians and parents are asked not to disclose which treatment they are using. Third, to minimize the risk of unmasking due to differences between emollients when applied to the skin (their look, feel or smell), parents are asked to maximize the amount of time between application and the assessment visits. Fourth, parents are also asked to ensure that the emollient container is hidden from view.

Researcher masking will be assessed using the Bang blinding index [15], which takes a value between −1 and +1: +1 indicates complete lack of masking and 0 is consistent with perfect masking. Negative values indicate that the respondent is wrong more often than would be expected by chance, which can arise, for example, if all participants are said to be on one particular treatment irrespective of what they receive. The index can be presented with confidence intervals and can be used as the basis of a test of the null hypothesis that the respondent is randomly guessing each participant’s allocation.

Routine clinical care is not affected – children will attend appointments for their eczema as normal and use other medications (for example, topical corticosteroids) as normally directed. If, during the course of the study, children have a reaction to the study emollient or parents wish to change for another reason, the clinicians in charge are free to prescribe any alternative as per their normal clinical practice. Co-prescribing of other leave-on emollients and bath additives will be discouraged, but allowed.

### Outcome measures

Participants will be followed up monthly (every 28 days) for 3 months (total 84 days). As this is a feasibility study, the primary outcome is the proportion of children approached who were randomized to a study emollient and used it for the duration.

Secondary outcome measures relate to the feasibility of the study:

- Data completeness of daily, weekly and monthly measures, recorded by parents and collected by research assistants.
- The extent to which the research assistants were kept masked to intervention.
- Preliminary data on the clinical effectiveness of the proposed study emollients, including the quantity and frequency of emollient application, and evidence of any effect on topical corticosteroid or calcineurin inhibitor use.
- Qualitative feedback from parents of participants regarding the logistics and acceptability of trial processes, procedures and paperwork.

### Data collection

How, when and by whom the data are collected are summarized in Table 1.

**Table 1:** Type, timing and means of data collected about participants in COMET study

Data collected	Frequency and timing of data collection				
	Baseline	Months 1–3			End of study
		Daily	Weekly	Monthly	
Medical history	R				
Eczema Area and Severity Index	R				
Six Area, Six Sign Atopic Dermatitis severity score	R				
Three Item Severity score	R				
Skin corneometry	R				
Study emollient use		P			
Other emollient or bath additive use		P			
Topical corticosteroid or calcineurin inhibitor use		P			
Patient-Orientated Eczema Measure	GP, PN or R		P		
Health care consultation attendance			P		
Eczema-related costs (out-of-pocket expenditure by parent)			P		
Time off work and nursery or day care due to eczema			P		
Dermatitis Family Impact	R			P	
Patient Global Assessment	R			P	
Quality of life	R			P	
Review of electronic medical record					GP or PN

GP, general practitioner; P, parent-completed daily diary; PN, practice nurse; R, research team member

#### Parent-completed daily diary

At enrolment, parents are given the option of completing a paper or electronic (app) version of the diary; and the option of automatic text reminders to encourage completion. They are asked to record use of emollient and topical corticosteroid or calcineurin inhibitor on a daily basis; detail contacts with healthcare professionals, eczema-related costs and the Patient-Orientated Eczema Measure [16] weekly; and complete Dermatitis Family Impact [17], Quality of Life [18] and global assessment questionnaires monthly.

#### Assessment visits

At the baseline visit, the researcher collects sociodemographic information, data on eczema diagnosis and treatments used, and asks the parents to complete the Dermatitis Family Impact questionnaire. At baseline and monthly follow-up visits, researchers undertake objective assessments of eczema severity (Eczema Area Severity Index; [19] Six Area, Six Sign Atopic Dermatitis severity score; [20] Three Item Severity score [21]) and measurements of skin hydration at the antecubital fossa and forearm (three measurements in each area) using a corneometer (Corneometer® CM825, Courage & Khazaka electronic GmbH, Cologne, Germany). Standardized procedures written in accordance with guidelines on biophysical skin measurements are followed [22].

At the final visit, parents are asked to complete an exit questionnaire. Parents who choose to withdraw from the study at any point are asked to complete a withdrawal questionnaire.

#### Review of electronic medical record

The primary care electronic medical records of consented children are reviewed for the 3 months they are in the study. Data on relevant prescriptions (emollients, topical and oral corticosteroids, topical calcineurin inhibitors) and health care use (number of consultations with GP or practice nurse and of dermatology out-patient attendances) are extracted.

### Data management

Parents of all participating children will be asked to provide consent using paper consent forms (Additional file 1). Consent forms and study questionnaires (case report forms) will be stored securely and made accessible only to the research team and authorized personnel.

Data from case report forms completed on paper by the participant or research team are entered in a study database, which incorporates data validation rules to reduce data entry errors, and management functions to facilitate auditing and data quality assurance. A random sample of 10% of diary and visit case report forms will be checked, by the trial research team, against entries in the database for quality purposes. At the end of the trial, the database will be cleaned and locked.

Patient identifiers will be kept in a separate system from the clinical data. The database and randomization system will be designed to protect patient information, in line with the UK Data Protection Act (1998). The research team will ensure that participants’ anonymity is maintained through secure handling and storage of patient information at the trial centre. Participants will be identified only by a patient ID number on the case report form.

### Analysis

Participant recruitment and follow-up via each of the two recruitment pathways will be reported using a CONSORT flowchart showing the numbers of people approached, eligible, recruited and randomized (with reasons for exclusions).

The proportion of children approached who were randomized to a study emollient and used it for the duration of the study (the primary outcome measure) will be reported. We will explore how participant recruitment and retention varies by recruitment pathway and practice and participant characteristics.

We will report data completeness of daily, weekly and monthly measures, recorded by parents and collected by the research team. In respect of the diary, we will compare data completeness between paper, with or without text reminders, and app versions. We will also, by referring to participants’ electronic medical record data, assess accuracy and completeness of parent-recorded diary data.

The different outcome measures will be presented as summary statistics, to allow sensitivity of each measure to change over time to be compared. The different emollients will be compared in terms of these summary outcome measures, and in terms of parent-completed questionnaires, to identify any early evidence of the inferiority of a particular emollient, which would inform the choice of emollients to be included in the main trial.

Feedback from parents of participants regarding satisfaction with the allocated emollient and trial processes, procedures and paperwork will be presented. Bang blinding index data will be presented to evaluate the success of keeping researchers masked to treatment allocation.

We will test the feasibility of using data collected during the trial to carry out a cost-effectiveness study from the perspectives of the NHS, parents, and the value of lost productivity. Data on resource use collected via the patient diary will be used to identify the level of missingness, by item, and which items are important cost drivers. This information will be used in designing data collection for the economic evaluation in the full trial. NICE recommends the use of quality-adjusted life years as the preferred outcome measure in economic evaluations. However, no validated generic measure of health-related quality of life is psychometrically and conceptually robust enough for young children under the age of 3. We are therefore using a preference-based measure of health in children with atopic dermatitis [18] in this feasibility study, which will indicate the value of using it to estimate quality-adjusted life years in the full trial.

### Sample size

Because this is a feasibility study, a formal sample size calculation for the estimation of treatment effectiveness has not be made. We will aim for a target sample size of 160, 40 in each arm. With this number, a true consent rate of 50% (160 children participating having invited 320 potentially eligible children) will be estimated, with a 95% confidence interval of the order 44% to 56%. This is sufficiently precise to inform the design of a definitive trial.

### Monitoring

The University of Bristol will act as sponsor for the trial (reference UoB2009). Because of the low risk nature of the study, it is being overseen by a joint Trial Steering and Data Monitoring Committee (TS/DM-C). The overall role of the TS/DM-C, which is independent of the sponsor, is to safeguard the interests of the trial’s participants, potential participants, investigators and sponsor; and to monitor the trial’s overall conduct, and protect its validity and credibility. No formal interim statistical analyses are planned: it is expected that the study recruitment will terminate when the intended sample size have been achieved. The committee will be masked to the identity of the treatment arms unless unexpected numbers of adverse events warrant examination of data by treatment allocation.

All adverse events will be recorded in participants’ study diaries. Expected adverse reactions to study emollients (for example, pruritus or erythema) will be collated and reviewed at Trial Management Group and TS/DM-C meetings. Expected non-serious (for example, upper respiratory tract viral infections, diarrhoea or vomiting) and serious (for example, lower respiratory tract infections or urinary tract infections) adverse events that cannot be causally related to study participation will not be reported. Expected serious adverse events that might be related to study participation will be reported to the sponsor within 24 hours of knowledge of the event. All relevant information about a suspected unexpected serious adverse reaction will be reported within 7 days to the Medicines and Healthcare Products Regulatory Agency (MHRA) and the ethics committee.

The chief investigator and study sites will allow monitors, persons responsible for the audit, representatives of the ethics committee and of the regulatory authorities to have direct access to source data and documents. Trial monitoring will be undertaken on behalf of the Sponsor by University Hospitals Bristol NHS Foundation Trust following their standard monitoring procedures.

### Ethics

The study was approved by the Central Bristol Research Ethics Committee (reference: 13/SW/0297), clinical trial authorization was given by the MHRA (reference: 03299/0017/001-003) and research governance approvals were obtained across all areas prior to recruitment. The study will be conducted in accordance with the principles of good clinical practice, UK Research Governance Framework and the Medicines for Human Use (Clinical Trial) Regulations 2006.

The current protocol version is 1.3. Version 1.0 (October 2013) was the version submitted for research ethics committee approval. Version 1.1 (November 2013) was the version first approved by both the research ethics committee and MHRA. Version 1.2 (May 2013) included minor wording and timeline changes and amendments to descriptions of study procedures, including adverse event reporting. Version 1.3 (November 2014) included revised eligibility criteria (from ‘child aged between 1 month and 3 years with doctor-diagnosed eczema’ to ‘child aged between 1 month and 5 years of age with eczema (diagnosed by a doctor or an appropriately qualified health care professional with oversight from a medically qualified doctor)’) and recruitment of additional sites. All amendments have been approved by the relevant regulatory bodies and communicated to relevant parties.

### Dissemination

Following a separate agreed publication policy, we will disseminate findings through local and national networks, including key professional, public stakeholder and educational organizations. Dissemination will also occur via oral presentations and posters to national primary care and dermatological scientific conferences, and by publication in peer-reviewed journals. For promotional and informative purposes, the study website [23] and on-line social media (Twitter [24] and Facebook [25]) are publically accessible. A summary of the main findings will be distributed via these routes at the end of the study and specifically for the participants involved.

## Discussion

This is the first study to determine the feasibility of a large trial to answer the research question, ‘What is the most clinically and cost-effective primary emollient to prescribe for young children with eczema?’ A feasibility study is necessary because there are a number of key uncertainties, including: optimal means of patient recruitment; the choice of interventions; feasibility of long-term patient-reported data collection; and the avoidance of bias in outcome measurement when parents cannot be kept masked from their children’s allocated treatments. In addition to establishing the feasibility of the definitive study, we are collecting data around the acceptability and effectiveness of four commonly used emollients, which may guide the choice of emollients in any future study.

During the first 7 months of recruitment, children had to be between 1 month and 3 years of age and have their eczema diagnosis confirmed by a doctor. Because of concerns about recruitment, we obtained permission to revise the eligibility criteria for the last 5 months of recruitment to: ‘Child aged between 1 month and 5 years of age with eczema (diagnosed by a doctor or an appropriately qualified health care professional with oversight from a medically qualified doctor)’.

Since the study was originally proposed, the HOME initiative has published welcome guidance on which measures of clinical signs should be included as a core outcome in clinical trials of patients with eczema (Eczema Area Severity Index) [26]. When the study was designed, there was uncertainty about which of the more commonly used means of assessing eczema severity should be used, hence the inclusion of three (Eczema Area Severity Index, Six Area, Six Sign Atopic Dermatitis severity score, Three Item Severity score) outcomes. However, it was always our intention to use this ‘duplication’ of effort to undertake secondary, methodological work, comparing the different measures and any relationship with the data on skin hydration we will obtain using the corneometer.

Despite this being a feasibility study of four of the emollients most commonly prescribed in the UK, which are also available to buy over the counter, COMET has been classed as a controlled trial of an investigational medicinal product in children. The associated regulation and accompanying paperwork and approval required have increased the set-up time and made study management more complex. Set-up was further complicated by the anomaly that in the UK two of the emollients are classed as medicines (Diprobase® cream and Doublebase® gel), one as a cosmetic product (Aveeno® lotion) and another as a medical device (Hydromol® ointment).

All participants are allocated an emollient (that is, there is no control group) because we believed that clinicians and parents would be reluctant to take part in a study where they might not be prescribed anything specifically to moisturize the skin; and because it would probably be deemed unethical to do so. It is possible that, depending on the acceptability and preliminary effectiveness findings, the number of emollients in the main trial could be reduced. It is not possible to mask carers and clinicians because the study emollients are very different in their consistency and smell, and because we are asking GP surgeries to prescribe the treatment.

At the end of the study, we will have established the feasibility of the main trial and produced valuable preliminary data on emollient acceptability and effectiveness. In the definitive trial, we will be able to compare the clinical and cost-effectiveness of the study emollients and in secondary analyses will be able to examine, for example, for evidence of a ‘steroid-sparing’ effect of emollients. As a consequence the treatment of children with eczema should be improved and costs to the NHS and patients reduced through fewer consultations and more efficient prescribing.

## Trial status

Recruitment started June 2014 and finished 30 April 2015.

## Additional file

Additional file 1:
**Participant consent form.**

## Abbreviations

COMET: Choice of Moisturiser for Eczema Treatment
CONSORT: Consolidated Standards of Reporting Trials
GP: general practitioner
MHRA: Medicines and Healthcare Products Regulatory Agency
NHS: UK National Health Service
NICE: National Institute for Health and Care Excellence
TS/DM-C: Trial Steering/Data Monitoring Committee
