# Supplementary material for: Choice of Moisturiser for Eczema Treatment (COMET): study protocol for a randomized controlled trial
Source: Trials. 2015 Jul 15;16:304. doi: 10.1186/s13063-015-0830-y (PMC4501045; doi:10.1186/s13063-015-0830-y)
Supplement: Additional file 1: — Participant consent form. [file 13063_2015_830_MOESM1_ESM.docx]

**Choice Of Moisturiser in Eczema Treatment (COMET)**Participant Consent Form

|  | Please initial box | |
| --- | --- | --- |
| 1. | I confirm that I have read and understand the participant information sheet dated 01.11.14 (version 1.3) for the above study. I have had the opportunity to consider the information, ask questions and have had these answered satisfactorily. | ⬜ |
| 2. | I understand that participation is voluntary and that we are free to withdraw at any time without giving any reason, without my child’s medical care or legal rights being affected. | ⬜ |
| 3. | I give permission for researchers working on this study to have access to my child’s records for the purposes of collecting information relevant to the aims of this study. | ⬜ |
| 4. | I give consent for the data collected in this trial to be used in future ethically approved studies on the understanding that all information will continue to be securely kept and remain confidential. | ⬜ |
| 5. | I understand that relevant sections of my child’s medical notes and all information collected for this research may be reviewed by researchers from the University of Bristol, from regulatory authorities or from the NHS Trust for the purpose of ensuring that the research is conducted appropriately. | ⬜ |
| 6. | I give consent to be contacted by a member of the research team with a view to being interviewed about my experiences of taking part in COMET. (I will be given more information first and I will be asked to give specific consent for this). | ⬜ |
| 7. | I agree to my child’s GP being informed of their participation in the study | ⬜ |
| 8. | I agree for my child to take part in the above study. | ⬜ |

_____________________________ ________________
Name of Participant Randomisation ID

__________________________ ______________ _______________

Name of Parent/Guardian Signature Date

__________________________ ______________ _______________

Name of person receiving consent Signature Date

Ensure that there are copies of the consent form for parent/guardian, GP site file, patient notes and one is faxed to the COMET team, as per the site agreement.

Office use only: PID _ _ _ _ Entered by _ _ _ on _ _ / _ _ / _ _
